# Teaching and assessment strategies in the obstetrics and gynaecology program: medical students' perceptions evaluation

**DOI:** 10.3389/fmed.2026.1861057

**Published:** 2026-09-25

**Authors:** Malak AlHakeem, Samy A. Azer, Mahmoud Salah Khalil, Sultan Ayoub Meo

**Affiliations:** 1 Department of Obstetrics and Gynaecology, College of Medicine, King Saud University, Riyadh, Saudi Arabia; 2 Department of Medical Education, College of Medicine, King Saud University, Riyadh, Saudi Arabia; 3 Department of Medical Education, College of Medicine, King Saud University, Riyadh, Saudi Arabia; 4 Department of Physiology, College of Medicine, King Saud University, Riyadh, Saudi Arabia

**Keywords:** assessment, gynaecology and obstetrics, medical students, program evaluation, teaching and learning

## Abstract

**Background:**

Worldwide, medical institutions face challenges in maintaining educational quality while accommodating the growing size of student cohorts. Assessment and critical analysis of educational programmes, particularly in the clinical years, are imperative for improving learning outcomes. This study aims to evaluate students' perceptions of the teaching and assessment methods used in the obstetrics and gynaecology programme.

**Methods:**

This observational, cross-sectional study was conducted among fourth-year medical students enrolled in the obstetrics and gynaecology programme at the College of Medicine, King Saud University, Riyadh. A total of 300 students were enrolled. An electronically generated questionnaire comprising four sections, including 19 closed questions and five open-ended questions in English, was used. The first section addressed responses on the course guide; the second and third sections evaluated students' perceptions of the various teaching and assessment methods; the fourth section collected generalised responses on students' preferences and suggestions for program improvement. The data were analysed in relation to the GPA of the students involved in the study.

**Results:**

Among the study participants, 46.2% of students were satisfied with the course programme outlines and timings, 34.2% with the clarity of the course objectives, 40.2% with the teaching methods, and 32.6% with the clarity of the assessment tools. The lowest mean agreement scores were recorded for the usefulness of skills lab sessions in achieving clinical objectives. There were significant differences between genders (*P* < 0.05). The highest percentages of Agree or Strongly Agree responses were for the appropriateness of the assessment tools for each course objective.

**Conclusion:**

This study shows that students were satisfied with the newly introduced teaching and assessment methods. However, student satisfaction rates do not appear to be related to student achievements. Moreover, satisfaction was lower regarding the course guide and the educational and assessment methodologies. The study highlights the need to review the clinical skills laboratory, its educational components, and students' tasks.

## Introduction

The field of medicine is competitive, and medical students are trained through a range of teaching strategies throughout their studies. Medical institutions worldwide face the challenge of maintaining educational quality while keeping pace with ongoing changes and improvements in medical practice. Assessment and critical analysis of educational programmes are crucial for determining whether educational goals have been achieved, ensuring alignment among teaching, learning outcomes, and assessment, and improving educational outcomes and the quality of graduates (1). Evaluation processes provide an opportunity to identify areas that need improvement and, at the same time, address these deficiencies and enhance educational practices (2). Since students' general perceptions of educational settings significantly influence their overall experience and are integral to the programme's success, these aspects must be carefully evaluated (2). Educational research has shown that students' insights and perceptions in the context of their learning behaviour tend to shape their learning behaviour, including motivation and learning, impact their study strategies, time investment, and overall academic outcomes, help in evaluating the learning environment and net satisfaction, and reveal misalignment between programme design and students' experience (3, 4).

The teaching and learning methods in medical schools must be designed to promote students' competency development as they progress through the program, leading to the desired outcomes. Different teaching institutions use various teaching/learning and assessment protocols to enhance students' achievement and satisfaction; it is crucial to identify the most effective methods, suit the students' learning needs, match the college environment, facilitate students' engagement, and enhance students' application of knowledge, problem-solving skills, and psychomotor and procedural skills (5).

At King Saud University, College of Medicine, we transformed the traditional 5-year programme into a student-centred, integrated hybrid problem-based learning (PBL) curriculum in the pre-clinical phase (the first two years). Teaching and learning focus on integrating basic biomedical sciences into system-based blocks, with students working in small groups to discuss PBL cases for each block. The curriculum introduces students early to clinical medicine through simulated patients and electronic cases, aiming to enhance psychomotor skills, knowledge application, and preparedness for clinical practice in the later years (6, 7). In the clinical years, the programme emphasises the expansion of patient-centred learning, hands-on clinical training, professionalism and professional practice, clinical competence, and patient safety. In addition, a research subject was introduced in years 2 and 3, with the aim not only of developing an understanding of research methods and methodologies, but also of enabling students, under supervision, to design and conduct a small research project with the potential for publication (8).

The gynaecology and obstetrics programme is delivered in the fourth year of the undergraduate medical curriculum. Recently, several elements of the programme design and its teaching and learning strategies were reviewed and refined. These included: (i) adoption of an outcome-based, competency-oriented educational framework; (ii) a spiral approach that revisits concepts introduced in the reproduction block in year 2 at progressively higher levels of complexity and clinical application; (iii) vertical and horizontal integration of basic sciences with clinical knowledge, ethical and professional principles, and clinical practice; (iv) emphasis on women-centred care, gender sensitivity, and effective communication skills; (v) experiential and workplace-based learning; and (vi) simulation-enhanced learning with a strong focus on patient safety. Constructive alignment between intended learning outcomes, teaching and learning activities, and assessment methods was a key design principle. Assessment strategies comprised scenario-based single-best-answer multiple-choice questions in the written examination to evaluate clinical reasoning, diagnostic decision-making, and management; Objective Structured Clinical Examinations (OSCEs) to assess clinical skills, communication, and patient safety; and workplace-based assessments using the Mini-Clinical Evaluation Exercise (9–12).

This study was designed to assess students' overall perceptions of teaching and assessment methodologies in the gynaecology and obstetrics program. The results can assist course organisers and the department of medical education in understanding the effectiveness of current educational practices, identifying areas in the program requiring improvement, evaluating alignment between teaching, learning outcomes, and assessment, and guiding evidence-based curriculum development and quality assurance. Our research question for this study was: How do 4th-year undergraduate medical students perceive the teaching and assessment methodologies used in the gynaecology and obstetrics program?

## Subjects and methods

### Study design, settings and sample size

This observational, cross-sectional study was conducted at the Department of Obstetrics and Gynaecology, College of Medicine, King Saud University, Riyadh, Saudi Arabia. The study participants were 4th-year medical students enrolled in the gynaecology and obstetrics programme. Students had the right to participate or not to participate in the study, and informed consent was obtained from each student before completing the online questionnaire. The sample size was calculated using the Yamane formula (13). The target sample size was 172; however, in this study, we recruited 184 fourth-year medical students.

### The study questionnaire

For this study, we reviewed the literature and developed an English language questionnaire. The questionnaire comprised 19 closed-ended and 5 open-ended questions in English. It was designed based on the Saudi MED competencies. All nineteen closed-ended questions were based on a 5-point Likert scale (1 = Strongly Disagree, 2 = Disagree, 3 = Not Sure, 4 = Agree, and 5 = Strongly Agree). The questionnaire comprised three sections. First section (number of items = 05): Students were instructed to indicate their unbiased opinions regarding the student course guide, which included four questions about the timing of the course guide's receipt and the clarity of the course objectives and assessments (1 = strongly disagree, 5 = strongly agree). The second section focused on teaching methods (number of items = 08). In the third section (number of items: 06), the students were instructed to describe their perceptions of the various teaching and assessment methods, using the same Likert scale.Two senior faculty members, medical educators, were invited to review the questionnaire items and check for any technical issues.

### Questionnaire validity

#### Content validity

The content validity of the questionnaire was established by mapping its items to the programme's objectives and aims, as well as to the Saudi MED Competency Framework (14). In addition, the questionnaire's subsections were aligned with the competencies, knowledge domains, psychomotor skills, and professional behaviours targeted by the programme and outlined in the Saudi MED framework.

Furthermore, content validity was assessed by reviewing the relevant literature and publications in leading medical education journals (15, 16). The two senior medical educators were invited to independently and anonymously review the questionnaire items, with particular emphasis on assessing alignment with core competency principles rather than subject-specific content (17).

#### Face validity

We conducted face validity testing to assess the extent to which the questionnaire appears to measure its intended construct. Although face validity has recognised limitations, it serves several important methodological and practical objectives. These include ensuring the clarity and comprehensibility of questionnaire items, assessing the perceived relevance and appropriateness of each item, and identifying potential flaws in question design (e.g., double-barrelled questions or internal contradictions). In addition, face validity testing helps detect weaknesses in item wording or question distribution that may affect participants' engagement or willingness to complete the questionnaire accurately. Collectively, these steps aim to enhance response rates and improve the overall quality of responses, as commonly addressed during piloting (17, 18). Face validity was established through independent review by experts in medical education and the subject area, the authors, and further refinement during the piloting process (19).

### Recruitment of study participants, data collection, and management

In our university hospital, the total number of medical students in the fourth medical class was 300; of these, 175 (58.3%) were male and 125 (41.7%) were female. For this study, we invited all 300 students; however, 184 (61.3%) responded to the survey and submitted the questionnaire in full. The sample size proportions were 71 (38.6%) males and 113 (61.4%) females.

A student leader was identified for each group to handle communications between the department and the students. These student leaders already fulfil the role of liaising between students and departments. The email addresses of these leaders were obtained from the college secretary. Group leaders were invited to a meeting to explain the nature of the project. Student group leaders were instructed to send the questionnaire to their group members.

The Google survey form was used to create our survey questionnaire for easy distribution to students and convenient return. Students were randomly assigned to equal groups. Completed survey responses were collected and downloaded using a Microsoft Excel 2016 worksheet (Microsoft Corporation, Redmond, Washington, USA). If Google Forms were used as stated earlier, meaning the questionnaire was available online, why then are groups and a leader needed? This raises several contradictory issues.

### Qualitative coding process for open-ended questionnaire items

The students' responses on the five open-ended question items were analyzed using thematic analysis. All responses were initially reviewed to gain an understanding of the data. After that, an inductive coding approach was employed, whereby important terms and statements were assigned initial codes based on their content. Similar codes were gathered into broader categories through a cyclic process, and these categories were subsequently organized into primary themes.

### Student's GPA scores

The Grade Point Average (GPA) of students were obtained via the questionnaire, confirmed by the assessment unit of the College of Medicine, KSU, and used as an indicator of academic performance. It was included in the analysis to explore its relationship with students' perceptions of teaching and assessment strategies in the Obstetrics and Gynaecology program. The GPA was treated as an independent variable and categorized into predefined ranges: high and low.

### Ethical approval and data analysis

The study was approved by the Institutional Review Board (IRB) of the College of Medicine, King Saud University, Riyadh (Ref # 18/0188/IRB). The data were analysed using SPSS version 23.0 (IBM Corp., Armonk, NY, USA). The descriptive statistics included frequencies and percentages for categorical variables and means and standard deviations for continuous variables. A chi-square test was used to compare categorical groups, and Student's t-test and ANOVA were used to compare continuous variables. Moreover, a regression analysis was performed to establish the association between GPA and students' level of agreement (satisfaction). A *p*-value < 0.05 was considered statistically significant.

## Results

In this study, approximately 300 students were enrolled; 125 (41.7%) were female, and 175 (58.3%) were male. A total of 184 fourth-year medical students responded to the survey (61.3%). There were 71 (38.6%) males and 113 (61.4%) females. The mean GPA for all respondents was 4.29 ± 0.53, with no statistically significant difference by gender (*P* = *0.851).*

### Student perception towards course guide

Table 1 presents the distribution of students' perceptions of the course guide and the teaching and assessment methods. It shows that more than half of students strongly agreed or agreed that the course guide was timely (46.2% and 15.2%, respectively), clear in explaining the course objective (34.2% and 24.5%, respectively), clear in explaining teaching methods (40.2% and 18.5%, respectively), and clear in explaining assessment tools (32.6% and 25.0%, respectively). Timely receipt of the course guide was the highest-scoring satisfaction item (x¯ = 3.63). The only significant gender difference was observed in satisfaction with the timely introduction of the course guide, which was higher among females than among males (*p* = .013).

**Table 1:** Mean levels of student agreement with student guide and teaching and assessment methods.

Main Item	Questions	Mean Score	Std. Dev
Student Guide	1. Received the course guide before the start of the course	3.63	1.552
	2. Objectives were clear and specific	3.52	1.422
	3. Methods of teaching were clear in the student course guide	3.59	1.464
	4. Assessment tools were clear in the student's course guide	3.48	1.426
Teaching Methods	5. Lectures were appropriate in covering the cognitive domain	3.57	1.440
	6. Videos were appropriate and related to teaching objectives	3.47	1.564
	7. Case discussions were appropriate and related to course objectives	3.49	1.500
	13. Case discussions were appropriate to apply your knowledge	3.54	1.444
	8. Skills lab covered all the relevant course clinical objectives	3.12	1.401
	9. Skills lab sessions helped achieve clinical objectives	3.07	1.391
	10. Clinical rotations were satisfactory in teaching	3.31	1.424
	18. Feedback and discussion were helpful in building experience	3.45	1.354
	19. Multimedia was helpful and appropriate for preparing the assignment and case presentation	3.55	1.409
Assessment Methods	11. Assessment tools were appropriate for each objective	3.43	1.401
	12. Written exams (MCQS) covered the cognitive objectives	3.35	1.429
	14. DXR helped assess the cognitive domains	3.03	1.554
	15. OSCE was appropriate for assessing the clinical (psychomotor) domain	3.46	1.305
	16. Mini-CEX was appropriate for assessing the clinical (psychomotor) domain	3.40	1.379
	17. Logbook suitable for assessing the clinical objective	3.28	1.447

Table 2 presents students' perceptions of teaching and assessment methods by gender and GPA. Results indicate that female students reported higher satisfaction with the course guide than male students, but the difference was not statistically significant. The estimated scores for males' and females' agreement about teaching methods were variable, but the gender difference was not statistically significant across all items. Similarly, Pearson's correlation coefficients between students' agreement with the course's teaching methods and GPA scores were estimated and showed no statistically significant associations. Pearson's correlation coefficients between the level of student satisfaction with the course guide and their GPA were also estimated but were not significant.

**Table 2:** Student perception score towards teaching and assessment methods according to gender and GPA.

Main Item	Questions	Female		Male		*P*-value (t-test)	GPA	
		Mean	Std. Deviation	Mean	Std. Deviation		Pearson Correlation	*P*-value
Timing and clarity of the student guide	1. I got the student guide at the appropriate time	3.72	1.623	3.49	1.433	.329	.047	.535
	2. Objectives were clear and specific	3.56	1.470	3.45	1.350	.614	.025	.743
	3. Methods of teaching were clear in the student guide	3.61	1.532	3.56	1.360	.827	.054	.478
	4. Assessment tools were clear in the student's guide	3.53	1.494	3.41	1.316	.561	.071	.345
Teaching methods	5. Lectures were appropriate in covering the cognitive domain	3.58	1.493	3.55	1.361	.904	.068	.366
	6. Videos were appropriate and related to teaching objectives	3.53	1.615	3.38	1.487	.518	.015	.846
	7. Case discussions were appropriate and related to course objectives	3.50	1.536	3.49	1.453	.991	.035	.642
	13. Case discussions were appropriate to apply your knowledge	3.50	1.524	3.61	1.315	.604	.075	.324
	8. Skills lab covered all the relevant course clinical objectives	3.23	1.395	2.94	1.403	.179	-.007	.921
	9. Skills lab sessions were useful in achieving clinical objectives	3.13	1.417	2.97	1.352	.442	.008	.921
	10. Clinical rotations were satisfactory in teaching	3.28	1.503	3.35	1.299	.742	.057	.451
	18. Feedback and discussion were helpful in building experience	3.44	1.445	3.46	1.205	.910	.064	.401
	19. Multimedia was helpful and appropriate for preparing the assignment and case presentation	3.53	1.458	3.58	1.338	.825	.053	.484
Assessment Methods	11. Assessment tools were appropriate for each objective	3.61	1.423	3.15	1.327	.029	.054	.477
	12. Written exams (MCQs) appropriately covered the cognitive objectives	3.35	1.510	3.35	1.299	.973	.078	.303
	14. DXR helped assess the cognitive domains	3.02	1.529	3.06	1.603	.872	.049	.514
	15. OSCE was appropriate for assessing the clinical (psychomotor) domain	3.41	1.386	3.55	1.169	.456	.040	.599
	16. Mini-CEX was appropriate for assessing the clinical (psychomotor) domain	3.45	1.433	3.31	1.294	.490	.064	.399
	17. Logbook suitable for assessing the clinical objectives	3.55	1.382	2.86	1.457	.002	.002	.982

### Student perception towards teaching methods

Table 3 presents the mean level of agreement with teaching methods (range: 3.07–3.57). The lowest rates of agreement and mean agreement scores were recorded for the usefulness of skills lab sessions in achieving clinical objectives (22.3% strong agreement, 16.8% agreement, and a mean score of 3.07). Rates of agreement with teaching methods tended to be higher among males than among females. Table 3 shows that the gender difference was significant for only two items: the appropriateness of videos and their relatedness to teaching objectives (*P* = .042) and the help of feedback and discussion in building experience (*P* = .018). Table 3 also shows that the level of agreement on teaching methods was higher among males than among females, except for the skills lab and clinical rotation items, where the pattern was reversed. A statistically significant difference between males and females was detected regarding the appropriateness of videos and their alignment with teaching objectives (*P* = .042) and the role of feedback and discussion in building experience (*p* = .018).

**Table 3:** Distribution of student Likert choices according to gender.

Main questions	Female			Male			*P-value* (Bonferroni corrected)
	Low	Medium	High	Low	Medium	High	
1. Received the student guide before the start of the course	22	9	61	10	12	24	.013
2. Objectives were clear and specific	14	10	44	9	13	19	.126
3. Methods of teaching were clear in the student guide	19	15	50	8	15	24	.300
4. Assessment tools were clear in the student's guide	17	14	44	9	13	16	.076
5. Lectures were appropriate in covering the cognitive domain	18	15	45	9	10	21	.385
6. Videos were appropriate and related to teaching objectives	24	11	50	13	5	20	.042
7. Case discussions were appropriate and related to course objectives	20	16	45	11	8	23	.440
8. Case discussions were appropriate to apply your knowledge	21	14	42	8	8	20	.160
9. Skills lab covered all the relevant course clinical objectives	15	30	32	15	20	14	.520
10. Skills lab sessions were useful in achieving clinical objectives	17	24	29	13	18	12	.576
11. Clinical rotations were satisfactory in teaching	20	16	35	5	16	19	.164
12. Feedback and discussion were helpful in building experience	18	25	38	5	14	15	.018
13. Multimedia was helpful and appropriate for preparing the assignment and case presentation	17	2	42	8	9	21	.228
14. Assessment tools were appropriate for each objective	15	15	42	10	15	13	.063
15. Written exams (MCQs) appropriately	19	15	38	7	16	17	.197
16. DXR helped assess the cognitive domains	29	19	28	19	10	21	.908
17. OSCE was appropriate for assessing the clinical (psychomotor) domain	14	21	35	4	20	18	.293
18. Mini-CEX was appropriate for assessing the clinical (psychomotor) domain	18	29	35	6	16	17	.106
19. Logbook suitable for assessing the clinical objectives	13	25	39	17	14	13	.029

#### Students' responses regarding teaching and assessment processes

Table 4 presents the valid codes from responses to open-ended questions. The highest numbers of codes were recorded for questions about areas for improvement in teaching and assessment methods, and for suggestions for improvement in the OB-GYN course (8, 9, and 8 codes, respectively). The first and second questions generated the fewest codes (4) for what was liked about teaching and assessment methods (7 codes). About the first question (Q1), four codes were identified as the most preferred teaching methods reported by students. The most preferred teaching method is videos (67.0%), particularly when used in conjunction with lectures. The next most preferred methods are tutor comment and group discussion, followed by clinical training (14.0% each). The least-preferred teaching method reported was lecture (9.0%).

**Table 4:** Students' responses to open-ended questions.

Questions	Pros of Teaching Methods (n) (%)	Pros of Assessment (n) (%)	Areas of Improvement: Teaching (n) (%)	Areas of Improvement: Assessment (n) (%)	Course Improvement Suggestions (n) (%)
1	Video (53) (67)	Prefer diversity and variation in evaluation (18) (32)	Replace lectures with video (14) (21)	Examiner unfair (7) (13)	Clinical skill exposure, especially for males (13) (21)
2	Clinical training (11) (14)	Mini-CEX (16) (28)	More improved videos (15) (23)	Change logbook; it is hard to cover (11)(21)	Video is to be improved (11) (18)
3	Group discussion is intriguing (11) (14)	Mark's distribution is fair (10) (18)	More clinical practice (16) (24)	DXR is not recommended (4)(8)	Lectures (different opinions) (6) (10)
4	Lectures primarily associated with videos (7) (9)	OSCE (8) (14)	Improve or remove DXR (5) (8)	MCQs need more time, weight (6)(11)	Logbook change or remove (9) (15)
5	-	Like nothing (7) (12)	Improve OSCE (2) (3)	Mini-CEX should be modified (7)(13)	Remove DXR (4) (7)
6	-	Video (7) (12)	More discussion (4) (6)	Videos need to be reviewed and validated (5)(10)	Good, no need for change (13) (21)
7	-		Change mini-CEX to be fairer (4) (6)	Questions to be modified (5)(10)	Bonus (3) (5)
8	-	MCQs are not the only way for evaluation (6) (11)	Modify logbook (6) (9)	It is fair; no need to improve (6)(12)	Others (4) (6)
9	-	-	-	Adoption of Bonus (3) (6)	-

n’= number of comments received (valid responses); ‘%’= the percentage of comments related to the code.

For the preferred methods of assessment (Q2), seven codes were identified. The most preferred code was diversity and variations in the assessment methods (32.0%). 28% of students considered mini-CEX a valid and effective evaluation method. It is worth mentioning that 18% of the respondents agreed that the distribution of marks on the course components was fair, and that 12% reported not liking any component of the assessment process.

Regarding Q1, Q2, and Q3, the students recommended improving the preferred methods mentioned in the first two questions. Increased clinical practice (24.0%), videos (23.0%), and replacing lectures with videos (21.0%) were the top recommendations for improving teaching methods, while changing the logbook (21.0%), modifying mini-CEX (13.0%), and advising examiners to be fairer (13.0%) were the most recommended ways to improve assessment methods. The most frequently suggested areas for improvement in the course were increasing clinical skills training, especially for male students (21.0%), followed by improved videos (18.0%) and changes or removals to the logbook (15.0%). In addition, the lower rates were reviewed and modified to reflect the removal of the logbook; OSCE, mini-CEX, and DXR were suggested to improve the course. A considerable percentage of students' responses indicated no need to modify the current course components (21.0%) or assessment methods (12.0%). In many cases, students commented that if the DXR or logbook cannot be modified or improved, they must be removed from the course components.

## Discussion

This observational, cross-sectional, institution-based study aimed to evaluate the upgraded teaching strategy introduced by King Saud University, College of Medicine, from students' perspectives. The study has evaluated students' perceptions of the various learning and assessment methodologies implemented and has described the relationship between students' satisfaction and academic achievement in the obstetrics and gynaecology course. This study shows that, in general, students were satisfied with the current teaching and assessment methods. However, student satisfaction rates do not appear to be related to student achievements. Additionally, student satisfaction was lower with the course guide, educational content, and assessment methodologies.

### Students' perceptions of teaching methodologies

More than half of the students in the present study agreed with the timings, clarity, teaching methods, and assessment tools. The following sections will discuss these perceptions individually, considering previously published data. One of the study's main goals was to assess students' perceptions of the course guide's timing and clarity. The course guide's timing and clarity are crucial to the learning process and to determining the efficacy of teaching strategies (20). From this perspective, providing a timely, clear course guide for medical students is an essential learning tool. Research revealed that students with high GPAs were satisfied with the course guide. They were also satisfied with the clarity of the objectives, teaching, and assessment methods. Similarly, a previous problem-based medical education study reported a higher agreement rate (93.9%) regarding the importance of a course guide in medical education (21). Using a course guide in clinical specialities may generate more interest than in basic sciences. The literature highlights that simulation-based teaching (22, 23) and bedside teaching and patient contact remain highly valued among Gynaecology and Obstetrics medical students (24).

It is essential to utilise an approach to teaching and learning that best suits students' needs. Assessing students' satisfaction with the teaching methods was a primary objective of this study. However, we found generally low to moderate agreement on the appropriateness of teaching methods. These findings disagree with a previous study by Narad et al., (25). This suggests we need to further improve these methods. Updating the course guide periodically with effective learning methodologies may help improve students' perception of the teaching methods. Faculty members continually consider improvements in learning outcomes, teaching methodologies, and innovation in their fields. The effectiveness of updated teaching methodologies can be periodically evaluated through student assessment and amended as needed (26). The low rate of agreement regarding the usefulness of the skill lab in this study contrasts with a previous study that found that students agreed or strongly agreed that a skill lab is a valuable tool for clinical learning.

According to a study, students prefer experiential learning methods, including role-playing with simulated patients and interacting with real patients in clinical settings (27). Incorporating these methodologies in our course can improve students' learning and satisfaction. This notion is supported by another finding from this study, which indicates relatively higher rates and scores for the clinical rotation. The difference in findings between this study and (28) can be explained by inconsistencies in the study guide, which relate to efforts to improve student satisfaction and facilitate learning. A clear definition of what constitutes an ‘acceptable' study guide must be stated and maintained throughout the samples, and when comparing with similar studies. Therefore, if the definition and quality of the study guide are inconsistent or unclear, it may warrant further investigation. Multimedia is another teaching method that has emerged as a powerful tool in recent years (29). This study highlighted the usefulness of this tool. The students' perceptions in this study align with previous findings. Multimedia is also helpful in teaching rare signs and cases through video lectures (30).

### Students' perceptions towards assessment methodologies

Assessment methods can help track students' learning and the effectiveness of teaching methodologies (31). The researcher aimed to assess the tools used for this purpose, including mini-CEX, logbooks, feedback and discussions, assignments, and case presentations using IT and multimedia. However, there was very little agreement on whether the various assessment methods were appropriate for the teaching process. This indicates that the assessment plan requires further improvement to meet medical students' expectations. Student opinions and satisfaction are critical in assessing teaching. They may play a significant role in monitoring, identifying strengths and weaknesses, and implementing necessary revisions to an educational programme (32).

Student satisfaction surveys can identify concerns about course shortcomings and provide opportunities for improvement, thereby enhancing the quality of teaching and learning. In this study, students evaluated teaching and assessment processes through open-ended questions, in addition to the traditional method that relies on closed questions with scoring, as mentioned above. Nearly the same results were obtained, but some new dimensions emerged from the open-ended questions: a preference for diversity and variation in assessment methods and a fair distribution of marks across the course components. These dimensions were incorporated into the closed questionnaire items. The researcher further conducted an in-depth analysis of the assessment methods used in this study. Diversity in teaching and assessment methods is thus generally preferred by students (33). No significant correlation between teaching or assessment methods and academic achievement was found in this study. This concurs with a previous descriptive-correlational study that also found no relationship between students' satisfaction and academic achievement (34).

#### Study strengths and limitations

This study has several advantages. It offers a thorough analysis of how medical students perceive the Obstetrics and Gynaecology programme's teaching and assessment methods, providing insightful information about the learning process. The reliability of the data collected is enhanced when a structured questionnaire is used. Additionally, the survey pinpoints specific areas of strength and opportunities for improvement, offering data that can guide curriculum development and enhance instructional strategies. The study's conclusions might add to the body of knowledge on medical education and be used as a guide in comparable learning environments. Similar to other studies, this study also has some limitations. First, the cross-sectional design, conducted at a single point in time, does not allow assessment of changes in perceptions over time or of causal relationships. Second, the findings are based on self-reported data, which may be subject to recall bias. Third, the study was conducted at a single institution, which may limit the generalisability of the findings to other medical schools with different curricula, teaching methods, or assessment practices.

## Conclusion

This study shows that, generally, students are satisfied with the newly introduced teaching and assessment methods. However, student satisfaction rates do not appear to be related to student achievements. Additionally, satisfaction was lower regarding the course guide and the educational and assessment methodologies. Differences in sampling, control definitions, content, types of medical education, and/or study types may explain the higher rates reported in other studies. Due to these discrepancies, further studies are needed during the clinical years of medical training, not only in Ob-Gyn courses but also in other surgical departments, while controlling for sampling and study design to accurately identify and improve educational and assessment methods in medical education in Saudi Arabia. The researcher suggests designing studies that assess and compare students' satisfaction rates across different medical domains, based on how frequently these domains undergo quality assurance checks, updates in teaching methodologies, and innovations in the field, to inform the design of teaching and assessment strategies suited to each domain.

## Data Availability

The raw data supporting the conclusions of this article will be made available by the authors, without undue reservation.
